# Global distribution, evolutionary dynamics, and origins of wheat streak mosaic virus

**DOI:** 10.3389/fpls.2025.1611008

**Published:** 2025-06-17

**Authors:** Dan Li, Xi Song, Fang Yang

**Affiliations:** School of Agriculture and Bioengineering, Longdong University, Qingyang, Gansu, China

**Keywords:** Wheat streak mosaic virus, evolutionary dynamics, global distribution, Bayesian phylogeographic reconstruction, diversification

## Abstract

Wheat streak mosaic virus (WSMV), one of the major pathogens affecting global wheat production, causes severe yield losses. Although its global diversification has been reported, the evolutionary dynamics and phylogeographic patterns of WSMV remain poorly understood. In the present study, we systematically investigated the global distribution of WSMV by integrating genomic sequences and literature reports. Furthermore, we analyzed the evolutionary dynamics and phylogeography of WSMV using 104 complete genomes and 218 coat protein (CP) gene sequences. Our results revealed that WSMV is currently spreading across 26 countries on six continents. Maximum likelihood (ML) phylogenetic analyses delineated four genotypes: Genotype I (Mexican lineage), Genotype II (Iranian-specific lineage), Genotype III (Eurasian-North American lineage), and Genotype IV (U.S.-Australian-Iranian lineage). Bayesian phylodynamic analysis estimated a mean evolutionary rate of 3.023 × 10^–4^ substitution/site/year (95% HPD: 1.945 × 10^-4^-4.187 × 10^-4^), and suggested that WSMV may have emerged in Iran, with the time to the most recent common ancestor (tMRCA) around 1700 (95% HPD: 1521-1850), although the relatively weak temporal signal limits precise timing (R² = 0.0585). Hierarchical tMRCA estimates revealed progressive diversification: Turkey and the United States at 1730 (95% HPD: 1580-1854), European countries at 1952 (95% HPD: 1915-1983), Kazakhstan at 1949 (95% HPD: 1911-1983), Australia at 1972 (95% HPD: 1953-1991), and Brazil at 1994 (95% HPD: 1981-2005). Bayesian phylogeographic reconstruction suggested Iran as a likely origin based on current data, with dispersal to the United States in the mid-19th century (1852-1887; Bayes factors (BF) = 5.26, posterior probability (PP) = 0.48) and to Turkey in the 20th century (1909-1942; BF = 65.76, PP = 0.92), both of which subsequently served as secondary hubs for global dissemination. The findings that WSMV has undergone persistent evolutionary diversification over extended temporal scales, and continues to spread globally, providing a framework for enhanced surveillance and control strategies.

## 1 Introduction

Wheat (*Triticum aestivum L.*), a cornerstone of global food security, serves as a primary staple crop for over one-third of the human population. It supplies approximately 20% of worldwide dietary energy and protein intake, with cultivation spanning 215 million hectares annually, underscoring its indispensable role in sustaining agricultural and nutritional systems. However, its productivity is continually jeopardized by a spectrum of viral pathogens, among which wheat streak mosaic virus (WSMV) emerges as one of the predominant biotic constraints on yield stability (Hasan et al., 2025). WSMV is a positive-sense, single-stranded RNA virus classified within the genus *Tritimovirus*, family *Potyviridae*. The genome is approximately 9.3 kb and encodes a single large polyprotein that is proteolytically cleaved into 10 mature proteins: P1, HC-Pro, P3, 6K1, CI, 6K2, VPg, NIa-Pro, NIb, and CP. P1 and HC-Pro are involved in polyprotein processing and suppression of host RNA silencing (Urcuqui-Inchima et al., 2001; Kasschau et al., 2003); P3 and CI contribute to viral replication and cell-to-cell movement (Carrington et al., 1998; Deng et al., 2015). 6K1 and 6K2 are membrane-associated proteins that aid in replication complex formation (Schaad et al., 1997; Grangeon et al., 2012); VPg is linked to the viral genome and plays a role in translation initiation (Murphy et al., 1996); NIa-Pro functions as a protease that cleaves the polyprotein into its functional units (Adams et al., 2005); NIb acts as the RNA-dependent RNA polymerase (Hong and Hunt, 1996). The WSMV coat protein (CP) gene, which encodes a conserved structural protein essential for virion assembly and vector-mediated transmission, serves a reliable molecular marker for phylogenetic analysis (Stenger et al., 1998; Tatineni et al., 2014; Robinson and Murray, 2013; Jones et al., 2022). Upon systemic infection of wheat, WSMV induces chlorotic leaf streaks that evolve into mosaic patterns, accompanied by severe stunting, premature senescence, reduced tillering, and malformed heads with shriveled grains, often leading to near−total yield loss under epidemic conditions (Singh et al., 2018). WSMV exhibits a global distribution with established presence in North America (Great Plains of the United States, Canadian Prairies), Europe (Russia, Ukraine, Germany), Oceania (eastern Australia), South America (Argentina, Brazil), Asia (Iran, Turkey, Kazakhstan), and Africa (South Africa), predominantly in temperate agroecological zones supporting intensive monoculture wheat production (Velandia et al., 2010; Bennypaul et al., 2019; Jones et al., 2022; Schubert et al., 2015; Kapytina et al., 2024; Nunna et al., 2025; Singh et al., 2018). In the United States, WSMV is most prevalent in the Great Plains, causing significant yield losses, particularly in Kansas, Nebraska, Colorado, Oklahoma, and Texas (French and Stenger, 2003; Wosula et al., 2017; Hadi et al., 2011; Jones et al., 2022). It imposes chronic yield suppression averaging 5% in Great Plains wheat systems, with episodic epidemics inducing localized crop failures exceeding 95% in high-risk counties under conducive agroecological conditions (French and Stenger, 2003; Robinson and Murray, 2013). Globally, WSMV has led to significant yield losses worldwide, exceeding 30% in key wheat-producing areas such as the Australian grain belt (Coutts et al., 2008) and Canada’s Prairie provinces (Bennypaul et al., 2019), underscoring its transboundary threat to food security.

WSMV is primarily transmitted via a persistent-propagative mechanism by its obligate vector, the wheat curl mite (WCM) (*Aceria tosichella Keifer*), which acquires virions during phloem feeding on infected hosts within a minimum acquisition access period (AAP) of 15 minutes. Viruliferous mites retain and transmit WSMV throughout their lifespan (3–4 weeks), enabling both horizontal (plant-to-plant) and vertical (transovarial) transmission (Gibson and Painter, 1957; Navia et al., 2013; Tatineni and Hein, 2018; Gupta et al., 2019). Epidemiologically, WCM-mediated spread occurs through two distinct pathways: (1) long-distance wind-aided dispersal of mites to hosts and (2) localized interplant transmission via physical contact between infected and healthy tissues (Coutts et al., 2008; Tatineni and Hein, 2018). While mechanical transmission via contaminated equipment contributes marginally to field infections (< 2%), seed transmission poses a critical biosecurity challenge. WSMV virions persist in wheat embryos and endosperm at rates of 0.1-2%, with viral RNA remaining detectable in seeds for up to 18 months under low-moisture storage, facilitating global dissemination through commercial seed networks (Jones et al., 2005; McKelvy et al., 2023; Hasan et al., 2025). Phylogenetic analyses of WSMV gene sequences suggest the virus was likely introduced to Australia via contaminated wheat seed, which originated from the Pacific Northwest region of the United States (Dwyer et al., 2007). Seed transmission rates of WSMV range from 0.2% to 0.5% across genotypes, with specific genotypes exhibiting up to 1.5% transmission efficiency. Despite these low rates, transboundary seed-mediated dispersal likely drives recombination events between geographically distinct WSMV strains, increasing viral genetic diversity and adaptive potential through the introduction of divergent genomic lineages into novel ecosystems (Jones et al., 2005; Singh et al., 2018).

Recent evolutionary analyses of global WSMV isolates using whole-genome sequences have uncovered significant phylogeographic divergence, delineating three major evolutionary clades (A, B, D) through a maximum likelihood (ML) framework. Clade A comprises a single Mexican isolate (GenBank No. AF285170). Clade B predominantly comprises representative isolates originating from Europe and Turkey. Clade D primarily comprises isolates from North and South America, Australia, and the Middle East, with U.S. isolates further diversifying into distinct regional subclades (Singh et al., 2018; Redila et al., 2021; Schubert et al., 2015). Due to the predominant representation of CP sequences in GenBank relative to whole-genome data, CP-based phylogenetic analyses provide a more comprehensive assessment of the global genetic diversity among WSMV isolates. Phylogenetic analyses based on the CP gene have identified four major WSMV clades (A, B, C, D), with clade C uniquely comprising isolates originating from Iran, a pattern not observed in whole-genome phylogenies (Singh et al., 2018; Vučurović et al., 2020). Jones et al. conducted CP gene-based phylogenetic analyses and classified WSMV strains into four phylogroups (I-IV), each corresponding to a distinct evolutionary lineage. Phylogroup I constitutes a genetically independent Mexican clade, while phylogroup II consists exclusively of Iranian strain clusters. Phylogroup III corresponds to strains within the Eurasian lineage, whereas Phylogroup IV encompasses a diverse assemblage of isolates from North and South America, Australia, and some Iranian strains (Jones et al., 2022). Although previous studies have documented WSMV evolution, comprehensive analyses integrating Bayesian evolutionary dynamics and phylogeographic inference remain limited. In this study, we systematically investigated the global distribution of WSMV, performed evolutionary analyses based on available whole-genome and CP gene sequences, and applied Bayesian phylogenetic and spatiotemporal modeling to elucidate the virus evolutionary dynamics. These results contribute to a deeper understanding of WSMV evolution and provide valuable insights for global surveillance and management strategies.

## 2 Materials and methods

### 2.1 Dataset preparation

All available WSMV complete genome (n = 104) (**Supplementary Table S1**) and CP gene (n = 114; > 700 bp) sequences were retrieved from GenBank (accessed 19 December 2024), and the final repository encompassed 218 CP gene sequences. Associated metadata, including accession No., country, strain name and collection date, were systematically extracted (**Supplementary Table S2**). To delineate the global distribution of WSMV, country-level occurrence records were analyzed through a dual-source framework: (1) genomic data from GenBank submissions (**Supplementary Table S3**), and (2) epidemiological reports catalogued in the CABI Digital Library (https://www.cabidigitallibrary.org/) for regions lacking sequence data but with documented WSMV outbreaks. A map of the global distribution of WSMV was created using QGIS 3.34.11 (QGIS, Geographic Information System. http://www.qgis.org).

### 2.2 Phylogenetic reconstruction

The WSMV genome sequences were aligned using MAFFT v7.520 (Katoh and Toh, 2008), followed by manual refinement of ambiguous regions in MEGA7. Nucleotide substitution model selection was conducted through ModelFinder implemented in IQ-TREE v1.6.12, where the generalized GTR+F+G4 demonstrated optimal fit based on Bayesian information criterion (BIC). ML phylogenetic reconstructions of complete genomes and CP gene datasets were performed in IQ-TREE v2.3.6, employing 1,000 ultrafast bootstrap replicates to quantify nodal support values. The trees were visualized using FigTree v1.4.4 and subsequently edited for publication-quality presentation in Adobe Illustrator.

### 2.3 Bayesian coalescent inference

Temporal signal analysis was conducted on ML phylogenies reconstructed from the WSMV complete genome and CP gene datasets using TempEst v1.5.3 (Rambaut et al., 2016). Sequences exhibiting significant outliers in the root-to-tip divergence versus sampling time regression were systematically excluded to enhance the chronological reliability of subsequent molecular clock calibrations. Time-scaled phylogenetic tree reconstruction was performed using BEAST v10.5.0 with BEAGLE library for computational acceleration (Drummond et al., 2002), implementing an uncorrelated lognormal relaxed molecular clock model selected through TempEst v1.5.3-based temporal signal validation. Nucleotide substitution model selection via ModelFinder (IQ-TREE v1.6.12) identified the generalized time-reversible model with empirical base frequencies and gamma-distributed rate heterogeneity (GTR+F+G4) as optimal under BIC minimization. To determine the optimal coalescent prior model among six candidate models (Constant Size, Exponential Growth, Logistic Growth, Expansion Growth, Hamiltonian Monte Carlo SkyGrid, Bayesian SkyGrid), we conducted comparative model selection through marginal likelihood estimation using path sampling (PS) and stepping stone (SS) methods. The Bayesian Markov chain Monte Carlo (MCMC) analysis was conducted with 400 million generations, sampling parameters and trees at 4000-step intervals. Convergence diagnostics were performed using Tracer v1.7.2 (Rambaut et al., 2018), where effective sample size (ESS) values exceeding 200 were considered statistically sufficient for all estimated parameters. Following the removal of initial 10% trees as burn-in to eliminate pre-convergence states, a maximum clade credibility (MCC) tree was reconstructed using TreeAnnotator v10.5.0 (BEAST package). The time-scaled phylogenetic tree was subsequently annotated and visualized through FigTree v1.4.4, with node bars representing 95% highest posterior density (HPD) intervals for divergence time estimates. A Bayesian coalescent framework implemented in BEAST v10.5.0 (Drummond et al., 2002) was employed to jointly infer the evolutionary rate (substitution/site/year, s/s/y), the time to the most recent common ancestor (tMRCA).

### 2.4 Bayesian phylogeography

Phylogeographic reconstruction was performed using discrete trait analysis in BEAST v10.5.0, implementing a symmetric state transition model to infer the spatial dynamics across geographic regions. MCMC was run for 400 million generations, with parameters logged every 40,00 steps and the first 10% of samples discarded as burn-in. ESS (> 200) was verified using Tracer v1.7.2. To statistically substantiate spatial diffusion patterns, a Bayesian stochastic search variable selection (BSSVS) approach was implemented to identify robustly supported migration routes under Bayes factors > 3. Bayes factors (BF) were derived from the resultant log files utilizing the spread.gl software, and the inferred migration routes were subsequently visualized within the same software (Li et al., 2024).

## 3 Results

### 3.1 Geographical distribution

**Figure 1:**
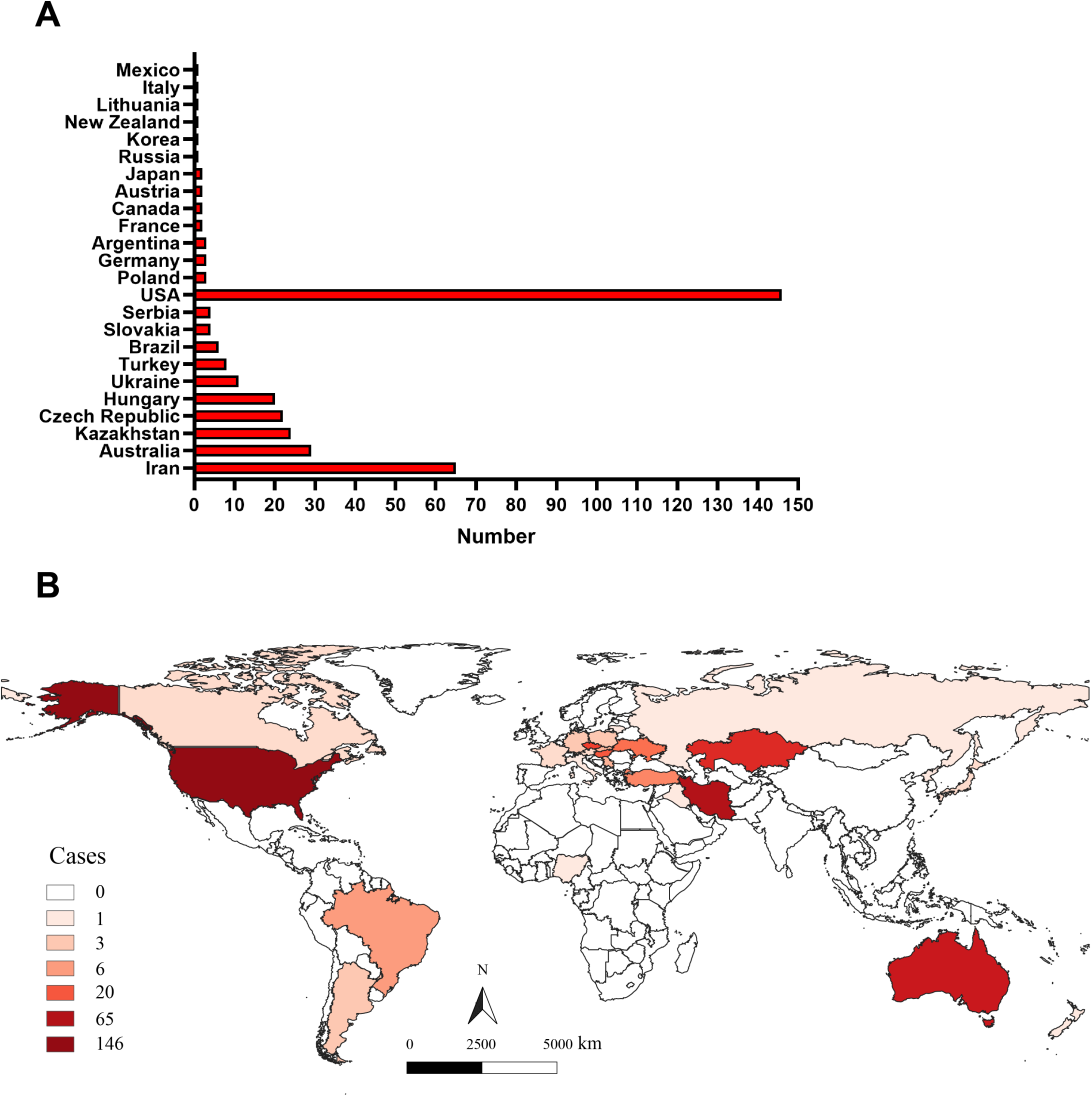
The global geographical distribution of WSMV. **(A)** The statistical analysis of the total number of WSMV gene sequences uploaded by all countries to the GenBank database. **(B)** The geographic distribution of WSMV, derived from the number of gene sequences submitted by various countries to the NCBI database, complemented by data from the CABI Digital Library (https://www.cabidigitallibrary.org/) and pertinent literature.

Although extensive literature has documented WSMV infection cases across various countries globally (Jones et al., 2022; Singh et al., 2018), a comprehensive analysis of its global distribution remains limited. In this study, we assessed the geographic distribution of WSMV based on the number of gene sequences uploaded by all countries in the GenBank (**Supplementary Table S3**), as well as data from the CABI Digital Library (https://www.cabidigitallibrary.org/) and relevant literature (Hasan et al., 2025). As shown in **Figure 1A**, as of December 19, 2024, gene sequences of WSMV have been uploaded from 24 countries to the NCBI database, with the United States contributing the largest number of virus strain sequences, totaling 146. Additionally, although Nigeria and Iraq have not uploaded gene sequences, literature has confirmed that WSMV has begun to spread in these two countries (Hasan et al., 2025). The analysis of the global geographical distribution of WSMV, based on the data provided above, reveals that the 26 countries reporting WSMV are distributed across six continents, including North America (USA, Canada, and Mexico), South America (Brazil and Argentina), Europe (Czech Republic, Hungary, Ukraine, Slovakia, Serbia, Poland, Germany, France, Austria, Lithuania, Italy, and Russia), Oceania (Australia and New Zealand), Asia (Iran, Kazakhstan, Turkey, Iraq, Japan, and South Korea), and Africa (Nigeria) (**Figure 1B**).

### 3.2 Phylogenetic analysis

**Figure 2:**
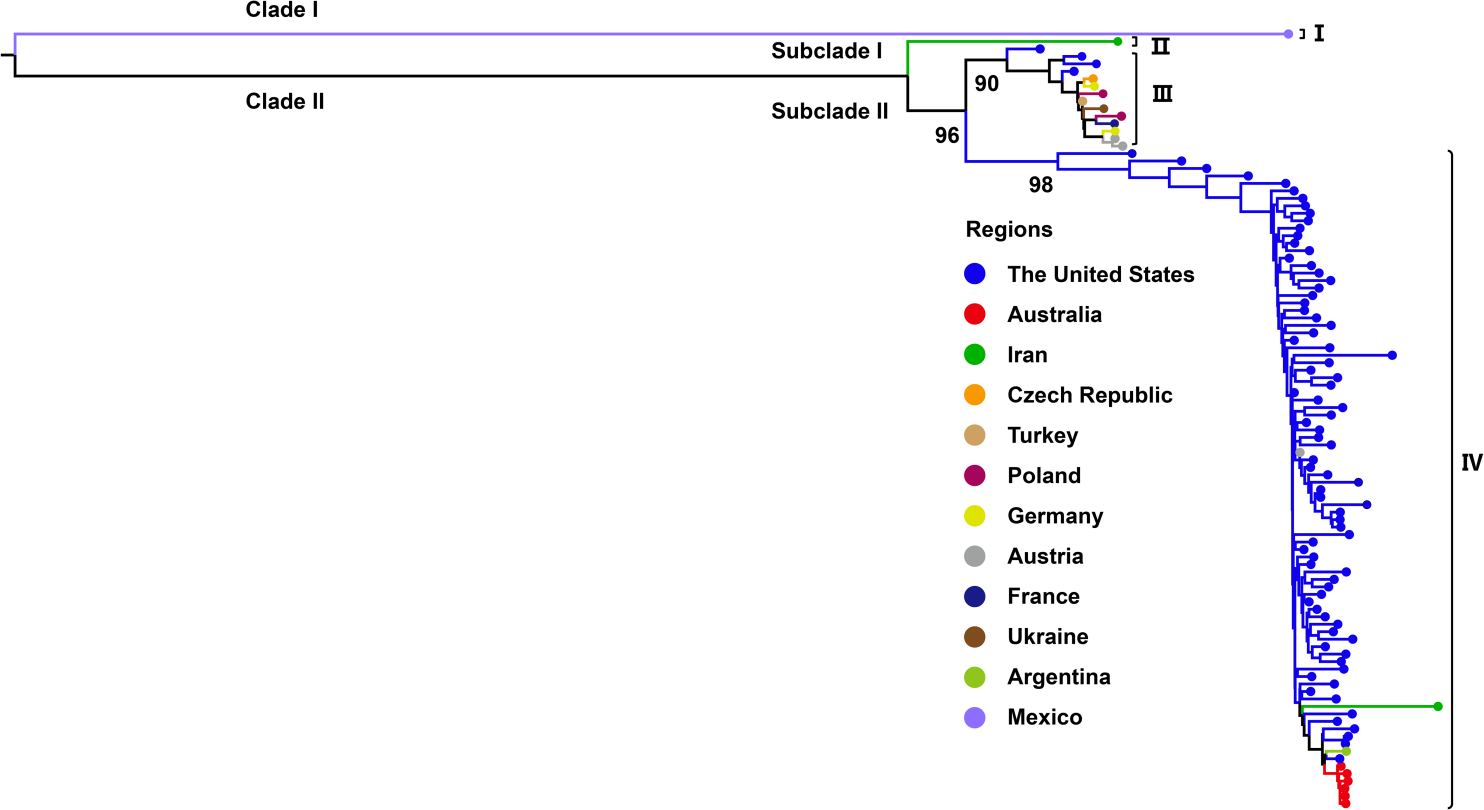
Maximum likelihood (ML) tree based on WSMV complete genome sequences (n = 104). ML phylogenetic tree was generated with IQ-TREE v2.3.6 using GTR+F+G4 model. Bootstrap analysis was performed with 1000 replicates. The trees were visu-alized using FigTree and edited employing Adobe Illustrator. The colors represent the virus collecting from different countries.

**Figure 3:**
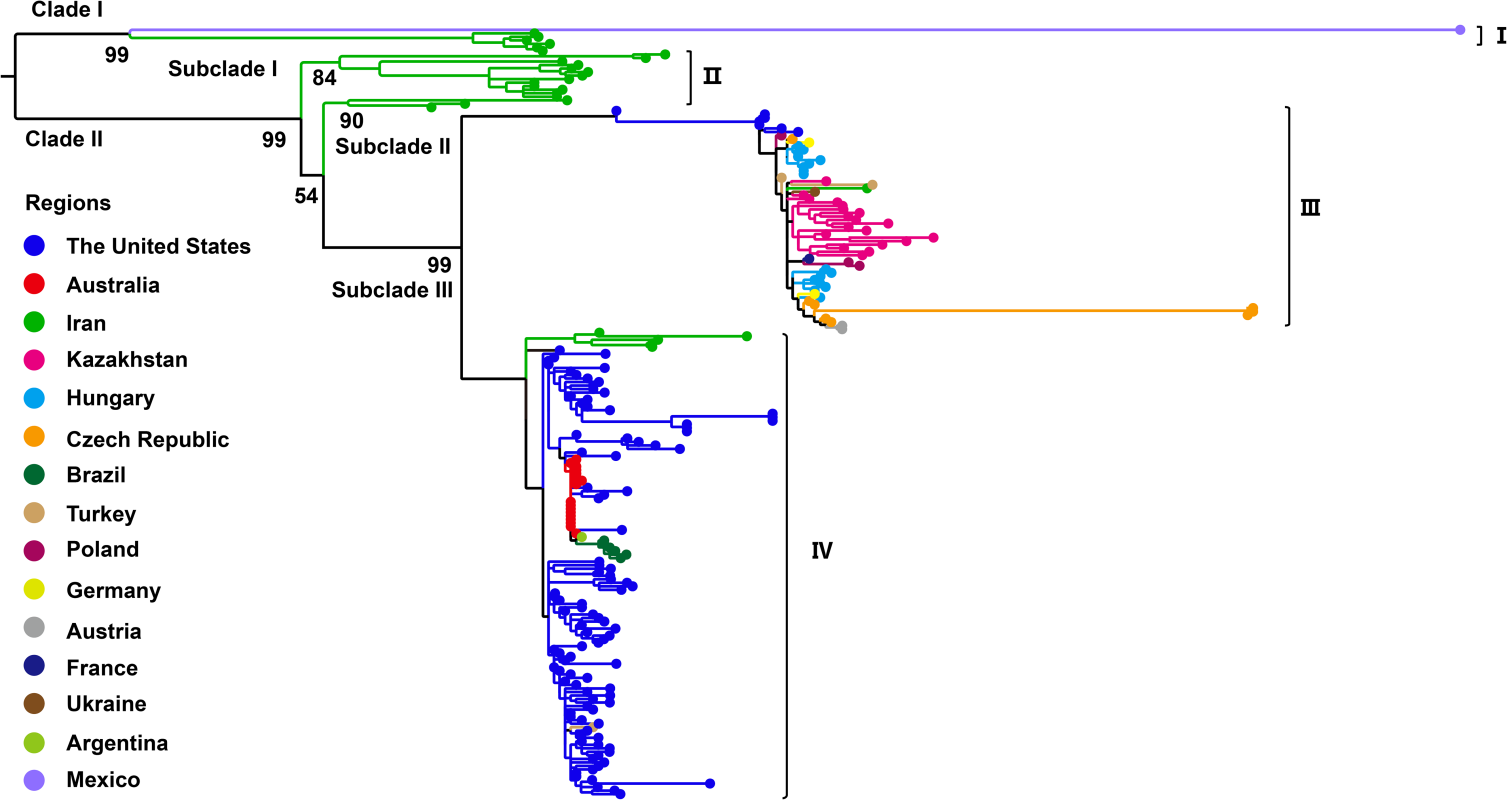
Maximum likelihood (ML) phylogenetic tree based on WSMV coat protein (CP) gene sequences (n = 218). ML tree was constructed with IQ-TREE v2.3.6 using GTR+F+G4 model. Bootstrap analysis was performed with 1000 replicates. The trees were visualized using FigTree and edited employing Adobe Illustrator. The colors represent the virus collecting from different countries.

Phylogenetic analysis based on the complete genome sequences of WSMV isolates using the ML method revealed a distinct bifurcation pattern, segregating all strains into two primary clades (Clade I and II) (**Figure 2**). Clade I, designated as genotype I, comprised exclusively of a single Mexican isolate (GenBank No. AF285170). Clade II was further subdivided into two subclades: Subclade I and Subclade II. Notably, Subclade I comprised only a single Iranian isolate (GenBank No. EU914918) and was classified as genotype II. In contrast, Subclade II was further divided into two well-supported lineages, corresponding to genotypes III and IV. The genotype III cluster predominantly contained European isolates from Germany, Czech Republic, Poland, Ukraine, France, and Austria, while maintaining distinct branches for American and Turkish isolates within this subclade. Genotype IV primarily consisted of U.S.-derived isolates, within which Australian strains formed a distinct subcluster, while also encompassing isolates from Iran, Argentina, and Austria (**Figure 2**). The topological structure of the phylogenetic tree constructed in this study is similar to previously reported ML phylogenies reconstructed from whole-genome datasets (Jones et al., 2022). Phylogenetic reconstruction using the CP gene classified all WSMV isolates into two primary clades (Clade I and II). Diverging from whole-genome phylogenetic patterns, Clade I displayed a bifurcated architecture consisting of a monophyletic subclade exclusively comprising Mexican isolates and a distinct Iranian-specific branch, collectively establishing this clade as Genotype I (**Figure 3**). Clade II further resolved into three subclades (subclade I-III), with subclade I and subclade II entirely composed of Iranian strains, designated as Genotype II. Subclade III underwent secondary divergence into two major lineages: Genotype III, predominantly containing European isolates alongside distinct sublineages from the United States, Kazakhstan and Turkey, and Genotype IV, primarily rooted in U.S. strains with nested clusters incorporating Australian, Brazilian, Argentine, and Iranian isolates forming independent branches (**Figure 3**).

### 3.3 Evolutionary dynamic analysis

To assess the temporal signal in the complete genome and CP gene datasets of WSMV, root-to-tip regression analyses were performed using TempEst, with sampling dates as temporal covariates. The complete genome dataset exhibited a negative regression slope (β = -4.0 × 10^-4^) and a low coefficient of determination (R² = 0.0157), indicating the absence of a statistically meaningful clock-like signal in the dataset. In contrast to the whole-genome analysis, the CP gene dataset exhibited a marginally positive temporal signal (β = 7.918 × 10^-4^, R² = 0.0585) (**Supplementary Table S4**), suggesting its potential utility for calibrating a relaxed molecular clock model despite suboptimal linear regression fit between root-to-tip divergence and sampling time. During the regression analysis of root-to-tip divergence, we excluded any sequence with an absolute residual > 0.01. Using these criteria, four outlier sequences (GenBank No. AF285170; KY419572; KY419573; KY419574) were identified as outliers and removed, resulting in a final CP gene dataset of 214 sequences for subsequent Bayesian evolutionary inference. To determine the optimal coalescent prior for demographic reconstruction, we systematically compared six candidate population models through marginal likelihood estimation using PS and SS methods. The logistic growth model demonstrated substantially higher log marginal likelihood values compared to alternative priors (**Supplementary Table S5**). Based on this statistical evidence, all subsequent phylogenetic analyses were conducted under the logistic growth coalescent prior coupled with a relaxed molecular clock model.

**Figure 4:**
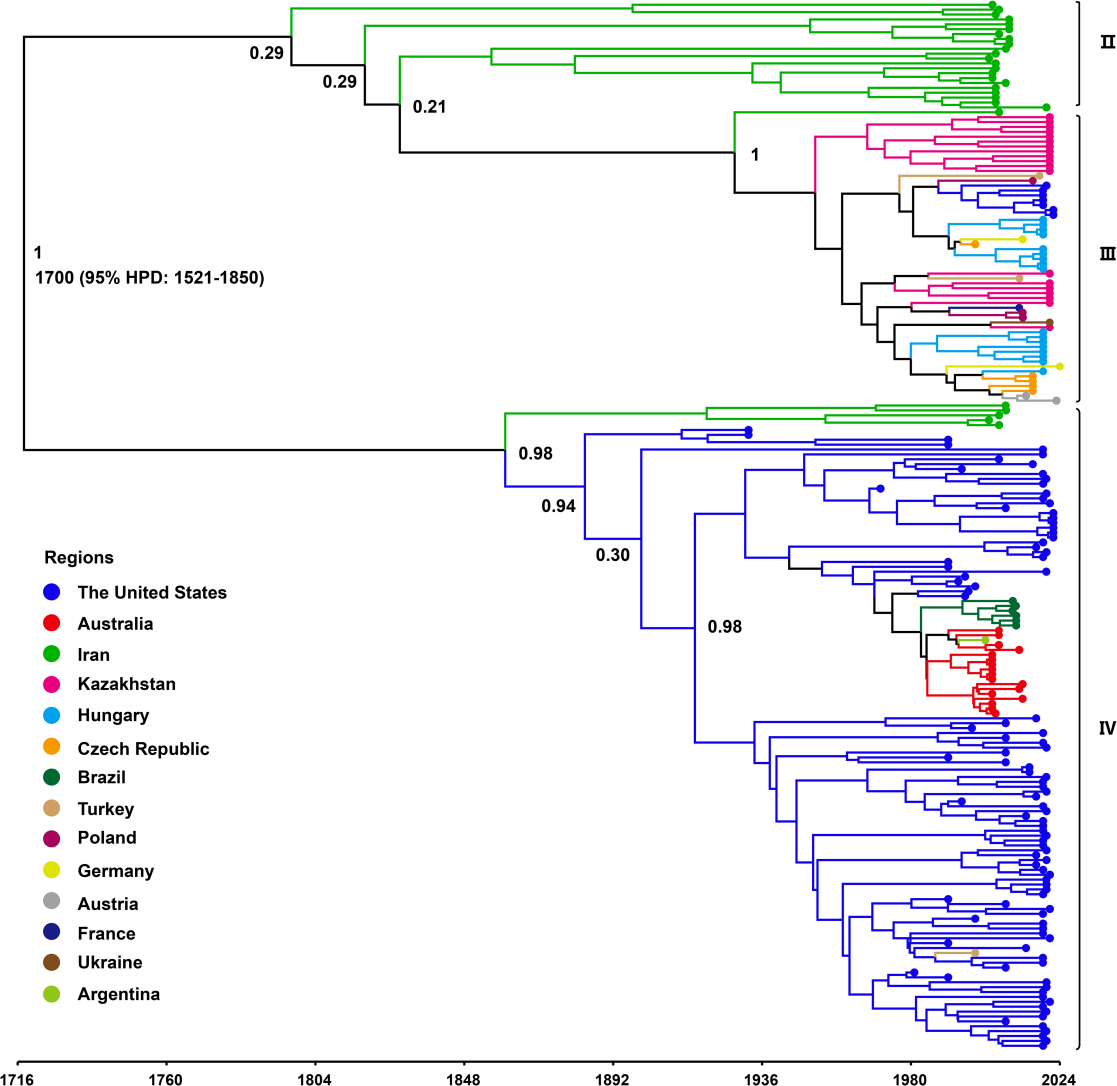
Time-scaled Bayesian phylogenetic tree based on WSMV CP gene sequences (n = 214). The colors represent the WSMV collecting from different countries. The posterior probabilities of the main nodes are shown.

To investigate the evolutionary dynamics of WSMV, a time-scaled phylogenetic tree was reconstructed using a Bayesian framework based on the CP gene. Following the removal of outlier sequences, the resulting tree topology demonstrated concordance with the ML phylogeny, resolving three distinct genotypes: Genotype II, comprising Iranian isolates; Genotype III, representing a Eurasian-American lineage; and Genotype IV, encompassing isolates from North America, Asia, and Australia (**Figure 4**). Following phylogenetic classification, Bayesian coalescent analysis was employed to estimate the evolutionary rate and reconstruct the temporal dynamics of WSMV dispersal. The mean substitution rate of WSMV was estimated at 3.023 × 10–^4^ s/s/y (95% HPD: 1.945 × 10^-4^-4.187 × 10^-4^), with the time to tMRCA estimated circa 1700 (95% HPD: 1521-1850) (**Table 1**). Based on this estimate and the early divergence of Iranian isolates, Iran is suggested as a likely geographic origin of WSMV, although the relatively weak temporal signal (R² = 0.0585) highlights some limitations in the temporal resolution of the dataset. From this origin, the subsequent spread of WSMV into Turkey and the United States (mean tMRCA: 1730; 95% HPD: 1580-1854) reflects the expansion of Genotype II into Eurasia and North America. The later divergence times in Kazakhstan (mean tMRCA: 1949; 95% HPD: 1911-1983) and Europe (mean tMRCA: 1952; 95% HPD: 1915-1983) indicate a stepwise geographic spread of Genotype II across continental regions. The relatively recent divergence of Australian (mean tMRCA: 1972; 95% HPD: 1953-1991) and Brazilian (mean tMRCA: 1994; 95% HPD: 1981-2005) isolates within Genotype IV suggests more recent, likely independent introductions of WSMV into these regions (**Figure 4**; **Table 1**). These findings provide key insights into the global evolutionary history and geographic structure of WSMV.

**Table 1:** Estimated time to the most recent common ancestor (tMRCA) of WSMV in major affected regions.

Country	Mean tMRCA	95%HPD	ESS
Iran	1700	1521-1850	260
The United States	1730	1580-1854	587
Turkey	1730	1580-1854	587
European	1952	1915-1983	482
Kazakhstan	1949	1911-1983	515
Australia	1972	1953-1991	1335
Brazil	1994	1981-2005	1269

### 3.4 Phylogeographic analysis

**Figure 5:**
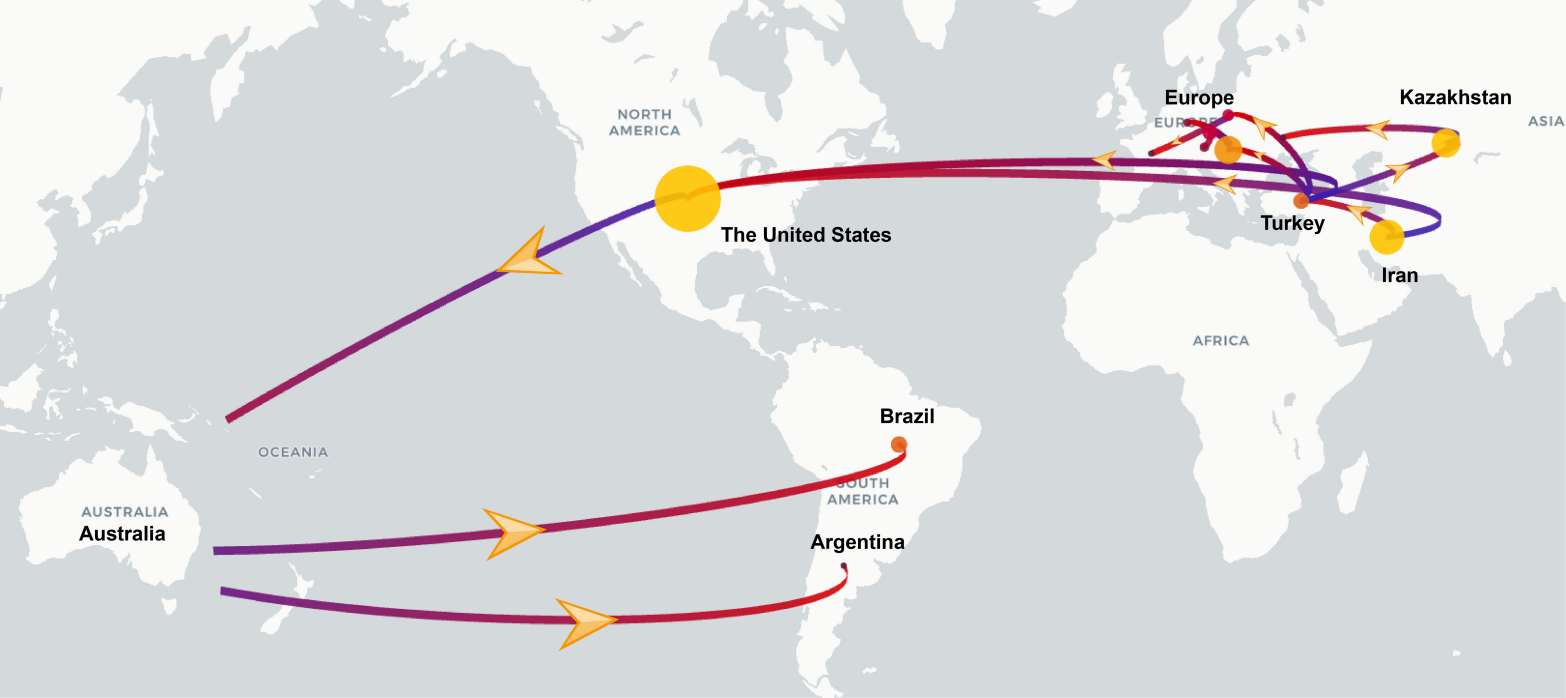
Discrete phylogeographic reconstruction of the spread of the WSMV worldwide. The start and end of the branches are coloured in blue and red, respectively. The size of the yellow clusters represents the cumulative lineage counts. Yellow arrows along the branches indicate the direction of viral spread. The global transmission routes of WSMV are displayed by filtering for Bayes factors greater than 5.

To investigate the global transmission routes of WSMV, we employed spread.gl to reconstruct the spatial transmission routes of WSMV. The results identified Iran as a likely origin of WSMV based on current data, with two inferred intercontinental dispersal trajectories: transatlantic spread to the United States (1852-1887; BF = 5.26, posterior probability (PP) = 0.48) and westward dissemination to Turkey (1909-1942; BF = 65.76, PP = 0.92) (**Figure 5**; **Table 2**). Turkey served as a crucial hub, facilitating the dissemination to Hungary (1942-1971; BF = 26.16, PP = 0.82) and Poland (1971-2016; BF = 15.38, PP = 0.73), ultimately contributing to its spread across various European countries. Simultaneously, Turkey played a mediating role in Asian incursions, notably facilitating the introduction of WSMV into Kazakhstan between 1955 and 1965 (BF = 16.23, PP = 0.74). The United States emerged as a main hub with dual origins: an initial introduction from Iran (1852-1887; BF = 5.26, PP = 0.48) followed by a subsequent resurgence originating from Turkey (1974-1995; BF = 65.76, PP = 0.92). This hub later mediated trans-Pacific transmission to Australia (1970-1979; BF = 35.2, PP = 0.86), from where it subsequently spread to South American countries, including Brazil (1979-1996; BF = 5.09, PP = 0.47) and Argentina (1992-2002; BF = 8.31, PP = 0.60) (**Figure 5**; **Table 2**).

**Table 2:** Well-supported WSMV transmission routes inferred from CP gene-based Bayesian phylogeographic analysis.

Data set	From	To	Start time	End time	Bayes factor	Posterior probability
WSMV CP gene	Iran	USA	1852	1887	5.26	0.48
	Iran	Turkey	1909	1942	7.43	0.57
	Turkey	Hungary	1942	1971	26.16	0.82
	Turkey	Kazakhstan	1955	1965	16.23	0.74
	Turkey	Poland	1971	2016	15.38	0.73
	Turkey	USA	1974	1995	65.76	0.92
	USA	Australia	1970	1979	35.20	0.86
	Australia	Brazil	1979	1996	5.09	0.47
	Australia	Argentina	1992	2002	8.31	0.60
	Poland	France	1983	2012	8.50	0.60
	Hungary	Czech	1987	1995	480.12	0.99
	Kazakhstan	Ukraine	2000	2021	33.48	0.86
	Czech	Austria	2005	2011	1157.09	1.00
	Hungary	Germany	1987	2024	95.63	0.94

## 4 Discussion

WSMV is recognized as a major pathogen threatening global wheat production (Gautam et al., 2023; Kapytina et al., 2024; Singh et al., 2018; Tatineni and Hein, 2023). The expansion of international trade and agricultural exchange has facilitated its transcontinental dissemination, with previous studies confirming its establishment across multiple countries spanning six continents (Velandia et al., 2010; Bennypaul et al., 2019; Jones et al., 2022; Schubert et al., 2015; Kapytina et al., 2024; Nunna et al., 2025; Singh et al., 2018). To improve our knowledge of the global distribution of WSMV, this study compiled all publicly available WSMV genomic sequences from GenBank and incorporated data from published reports documenting its presence in regions where genomic data remain unavailable. The results indicate that WSMV is currently detected in 26 countries across six continents: North America (USA, Canada, Mexico), South America (Brazil, Argentina), Europe (Czech Republic, Hungary, Ukraine, Slovakia, Serbia, Poland, Germany, France, Austria, Lithuania, Italy, Russia), Oceania (Australia, New Zealand), Asia (Iran, Kazakhstan, Turkey, Iraq, Japan, South Korea), and Africa (Nigeria). Although seed transmission of WSMV remains low (documented at only 0.5-1.5%), the potential risk posed by this pathogen should not be overlooked (Jones et al., 2005; Lanoiselet et al., 2008). The introduction of genetically distinct WSMV strains into new environments may facilitate recombination events, potentially giving rise to more virulent variants (Singh et al., 2018; Muhammad et al., 2022; Ilbağı et al., 2024). Consequently, major wheat-producing and -importing countries should prioritize coordinated management strategies, including enhanced surveillance, deployment of resistant cultivars, and strengthened phytosanitary regulations to prevent further spread and evolution of WSMV.

Previous phylogenetic analyses of WSMV using the full genome sequences have classified global isolates into three clades (A, B, and D), corresponding to a single Mexican strain, European-Turkish lineage, and North/South American-Australian-Turkish lineage, respectively (Schubert et al., 2015). In the present study, our whole-genome-based phylogenetic analysis delineated four genotypes (I-IV). Genotypes I, III, and IV generally align with these previously defined lineages, representing the independent Mexican lineage, the Eurasian-North American lineage, and the U.S.-Australian-Turkish-Iranian lineage, respectively. Notably, Genotype II comprises an independent Iranian strain, which is consistent with the recent study (Redila et al., 2021). Phylogenetic analysis based on the CP gene sequence divides the WSMV population into four clades (A, B, C, and D) along with a recently introduced clade, B1 (Singh and Kundu, 2017; Vučurović et al., 2020). Additionally, a recent study divided WSMV into four phylogroups (I-IV) based on CP gene analysis, generally corresponding to different evolutionary lineages: a single Mexican lineage, Iranian-specific lineage, Eurasian-North American lineage, and U.S.-Australian-Iranian lineage. Our CP gene-based results are largely consistent with those groupings, further validating our genotype designations (I–IV) across both complete genome and CP gene datasets. Importantly, our study incorporated newly available sequences and applied strict quality thresholds (CP gene >700 bp), which improved resolution but excluded some low-quality or partial sequences. Additionally, time-calibrated phylogenetic reconstructions in this study further revealed that even within the same genotype, WSMV from the same country, such as the United States, Iran, and Kazakhstan, have clustered into distinct sub-lineages. The observed phylogenetic diversity and geographic structuring of WSMV align with broader evolutionary patterns documented within the *Potyviridae* family, which is characterized by high genetic variability and pronounced lineage differentiation (Mbanzibwa et al., 2011; Gao et al., 2022; He et al., 2022). The broad host range of the virus, which includes wheat, maize, and various wild grasses, likely facilitates host-associated selective pressures that drive genetic diversification, a fundamental process underlying potyvirus evolution (Jones, 2021; Jones et al., 2022). In addition, transmission by the WCM may impose genotype-specific transmission bottlenecks, further shaping population structure (Nachappa et al., 2021; Pingault et al., 2024). WSMV is frequently found in mixed infections with other cereal viruses such as Triticum mosaic virus (TriMV), High Plains wheat mosaic virus (HPWMoV, also known as High Plains virus, HPV) and Barley yellow dwarf virus (BYDV), which can modulate host immune responses and replication conditions (Nunna et al., 2025; Pozhylov et al., 2022). These co-infections may increase within-host viral diversity and enhance WSMV fitness through synergistic interactions, thereby accelerating the emergence of novel variants under intensified agricultural practices. Collectively, our data underscore the multifactorial nature of WSMV evolution and highlight the importance of integrating genomic, ecological, and epidemiological data to support effective surveillance and management strategies.

WSMV was first documented in 1922 as a plant pathogen causing “yellow mosaic” symptoms in wheat in Nebraska, within the Central Great Plains of the United States (McKinney, 1937; Staples and Allington, 1956). Over the past century, extensive research has underscored its global epidemiological significance as one of the most persistent and genetically diverse viruses impacting wheat (Singh et al., 2018). However, the origin of WSMV both temporally and geographically remains a subject of debate, with limited studies addressing this issue. Uncertainties persist regarding whether the WSMV originated in the Old World or the New World, and whether its source can be traced to a single Mexican strain or to the West Central Eurasia (Jones et al., 2022). Jones et al. employed complete genome sequences of WSMV and their collection dates to estimate tMRCA, which was inferred to be 1456 (95% HPD: 817-1690), with the divergence of phylogroups III and IV estimated to have occurred around 1829 (95% HPD: 1700-1910) (Jones et al., 2022). In the present study, we also attempted to estimate the tMRCA using complete genome sequences and sampling dates; however, the root-to-tip regression analysis yielded a negative slope, indicating statistically insignificant temporal signal for molecular clock calibration. Consequently, tMRCA estimation was conducted using the CP gene dataset, which exhibited a marginally positive temporal signal (β = 7.918 × 10^-4^, R² = 0.0585). Although the temporal signal was weak, the CP gene dataset provided the most comprehensive temporal and geographic coverage available. To accommodate rate heterogeneity among lineages, a relaxed molecular clock model was implemented. While the limited clock-likeness of the dataset may introduce some degree of uncertainty in estimating evolutionary rates and divergence times, the analysis nonetheless yielded meaningful insights. Specifically, our results estimated the tMRCA of WSMV to be approximately 1700 (95% HPD: 1521-1850), partially overlapping with the 95% HPD interval reported by Jones et al., thereby supporting the plausibility of their earlier inference (Jones et al., 2022). Additionally, our Bayesian evolutionary analysis estimated a mean substitution rate of 3.023 × 10–^4^ s/s/y (95% HPD: 1.945 × 10^-4^-4.187 × 10^-4^). To further elucidate the potential origin of WSMV, we estimated the tMRCA for isolates from various countries. The results revealed that Iran exhibited the earliest mean tMRCA (1700; 95% HPD: 1521-1850), followed by Turkey and the United States (mean tMRCA: 1730; 95% HPD: 1580-1854), Kazakhstan (mean tMRCA: 1949; 95% HPD: 1911-1983), and European countries (mean tMRCA: 1952; 95% HPD: 1915-1983). More recent divergence times were observed for Australia (tMRCA: 1972; 95% HPD: 1953-1991) and Brazil (tMRCA: 1994; 95% HPD: 1981-2005). These findings further indicate that the estimated tMRCA for each country predates the corresponding year of official detection or reporting, implying that WSMV may have been present and circulating undetected for a considerable period before its formal identification (McKinney, 1937; Staples and Allington, 1956; Bremer, 1971; Bennypaul et al., 2019; Sánchez-Sánchez et al., 2001; Mar et al., 2013; Stenger and French, 2009; Jones et al., 2022; Vučurović et al., 2020). The relatively broad HPD intervals observed, particularly for early-diverging regions such as Iran, may be attributed to multiple sources of uncertainty, including sparse historical sequence data, limited temporal resolution due to weak molecular clock signal, and the potential influence of recombination among viral genomes. In this study, recombination analysis using RDP4 identified three potential recombinant sequences (data not shown). Given their limited number and negligible influence on the overall phylogenetic topology, these sequences were retained to ensure analytical consistency and maximize geographic representation. Although these factors may reduce the precision of tMRCA estimates, the CP gene dataset remains the most temporally and geographically representative resource currently available for WSMV. As such, the inferences drawn from this analysis, though subject to inherent limitations, offer valuable insights into the virus’s evolutionary history. Furthermore, the results support the hypothesis that WSMV most likely originated in the Old World, with Iran representing a putative center of origin based on its earliest estimated tMRCA. The relatively early divergence times observed in Turkey and the United States suggest that these regions acted as key secondary hubs in the early intercontinental spread of the virus.

To validate the hypothesis of an Old World origin and elucidate global transmission routes, we performed Bayesian phylogeographic reconstruction using the spread.gl software. Our results suggest Iran as the likely source of initial WSMV dissemination based on current data, with inferred transmission routes to the United States (1852-1887; BF = 5.26, PP = 0.48) and Turkey (1909-1942; BF = 65.76, PP = 0.92). Turkey subsequently emerged as a key hub for intercontinental spread, with strong support for onward transmission to Hungary, Poland, and Kazakhstan. Notably, our analysis identified two distinct introduction routes into the United States: one directly from Iran in the 19th century (1852-1887; BF = 5.26, PP = 0.48), and another from Turkey in the late 20th century (1974-1995; BF = 65.76, PP = 0.92), with only the latter receiving strong support. These temporally staggered introductions provide a novel phylogeographic insight, suggesting that multiple independent events contributed to shaping the global evolutionary trajectory of WSMV. Further spread occurred from the United States to Australia (1970-1979; BF = 35.2, PP = 0.86), and from Australia to South America, including Brazil and Argentina. These long-distance movements likely reflect historical seed exchange, expanding wheat trade, and unintentional transboundary transport of infected planting materials (Jones, 2021). The results underscore the importance of considering complex and asynchronous pathways that involve repeated introductions and multiple dissemination hubs in understanding the global spread of plant viruses.

In conclusion, our results determined that WSMV has achieved global distribution across 26 countries on six continents, exhibiting global diversity driven by adaptive variations in different regions. Furthermore, Bayesian evolutionary reconstruction revealed that WSMV likely originated in Iran around 1700, based on available evidence, although the weak temporal signal introduces some uncertainty to the exact timing. Subsequent global spread appears to have been facilitated by transmission hubs such as Turkey and the United States. These findings enhance our understanding of the global evolutionary dynamics of WSMV and provided valuable insights for its effective management and control.

## Data Availability

All sequence data used in this study were retrieved from GenBank. Accession numbers for all sequences are provided in the **Supplementary Materials**. No new sequence data were generated.
