# Supplementary material for: Global distribution, evolutionary dynamics, and origins of wheat streak mosaic virus
Source: Front Plant Sci. 2025 Jun 17;16:1611008. doi: 10.3389/fpls.2025.1611008 (PMC12209379; doi:10.3389/fpls.2025.1611008)
Supplement: Supplementary file 1 [file SupplementaryFile1.zip › Sup Table 4 and 5.docx]

**TABLE S4** Root-to-Tip regression parameters for WSMV complete genome and CP gene sequences analyzed using TempEst

| Index | WSMV complete gene | CP gene |
| --- | --- | --- |
| Slope （β） | -4.0 × 10^-4^ | 7.918 × 10^-4^ |
| Correlation Coefficient | -0.1255 | 0.2418 |
| R^2^ | 1.5746 × 10^-2^ | 5.845 × 10^-2^ |

**TABLE S5** Marginal likelihood estimates of six coalescent prior models using path sampling (PS) and stepping stone sampling (SS) methods

| Site Model | Clock Model | Coalescent prior | Path sampling | Stepping stone sampling |
| --- | --- | --- | --- | --- |
| GTR+F+G4 | Uncorrelated lognormal relaxed molecular clock | Constant Size | -9583.28 | -9585.15 |
|  |  | Exponential Growth | -9575.58 | -9576.75 |
|  |  | Logistic Growth | -9352.29 | -9354.49 |
|  |  | Expansion Growth | -9573.70 | -9574.70 |
|  |  | Hamiltonian Monte Carlo SkyGrid | -9549.90 | -9553.80 |
|  |  | Bayesian SkyGrid | -9555.77 | -9557.12 |
